# Mechanical and Biological Properties of Titanium and Its Alloys for Oral Implant with Preparation Techniques: A Review

**DOI:** 10.3390/ma16216860

**Published:** 2023-10-25

**Authors:** Haochen Wu, Xiaohong Chen, Linghui Kong, Ping Liu

**Affiliations:** School of Materials and Chemistry, University of Shanghai for Science and Technology, Shanghai 200093, China; tonywu0713@outlook.com (H.W.); cxh992@163.com (X.C.); konglinghui2022@163.com (L.K.)

**Keywords:** titanium, dental implant, osseointegration, antibacterial, osseoperception

## Abstract

Dental implants have revolutionised restorative dentistry, offering patients a natural-looking and durable solution to replace missing or severely damaged teeth. Titanium and its alloys have emerged as the gold standard among the various materials available due to their exceptional properties. One of the critical advantages of titanium and its alloys is their remarkable biocompatibility which ensures minimal adverse reactions within the human body. Furthermore, they exhibit outstanding corrosion resistance ensuring the longevity of the implant. Their mechanical properties, including hardness, tensile strength, yield strength, and fatigue strength, align perfectly with the demanding requirements of dental implants, guaranteeing the restoration’s functionality and durability. This narrative review aims to provide a comprehensive understanding of the manufacturing techniques employed for titanium and its alloy dental implants while shedding light on their intrinsic properties. It also presents crucial proof-of-concept examples, offering tangible evidence of these materials’ effectiveness in clinical applications. However, despite their numerous advantages, certain limitations still exist necessitating ongoing research and development efforts. This review will briefly touch upon these restrictions and explore the evolving trends likely to shape the future of titanium and its alloy dental implants.

## 1. Introduction

The teeth are the most complex organ in the human body [1]. They can chew food, aid in pronunciation, and beautify the features. Tooth defects will affect normal occlusion, plug the tooth, and cause food residue accumulation [2], while tooth loss will cause gingival atrophy, displacement of adjacent teeth, reduced chewing function, influence pronunciation and appearance, etc [3]. According to the fourth National Oral Epidemiology Study, the overall incidence of oral diseases in China was over 90%. There are different treatment options for various types of tooth damage. If the damaged area of the tooth is limited or the extent of damage, while significant, allows for repair, the appropriate choice would be dental filling [4]. A dental crown restoration is recommended in cases where there is a substantial defect in the tooth crown, but the root remains intact, and neighbouring teeth are healthy [5]. The suitable option would involve a dental prosthesis where the tooth is lost and no root structure exists [6]. There are three main types of dental prostheses: removable dentures [7], fixed dentures [8], and implants [9]. While removable dentures are affordable and can be taken out [10], they can impact aesthetics and pronunciation and lead to a sensation of a foreign object in the mouth. Fixed dental prosthetics exhibit robust chewing functionality and eliminate the necessity for daily removal and cleansing [11]; however, they can harm adjacent teeth. Dental implants have attracted wide attention because they can prevent alveolar bone atrophy and absorption without damaging the surrounding dental tissue and have a vital chewing function [12]. Evidencing their significance, in 2022, the global implant and denture market reached USD 10.47 billion and is expected to reach USD 11.54 billion in 2023 [13].

The realm of implant materials encompasses various options, including metal [14], ceramic [15], polymer [16], etc. The ceramic colour is beautiful, but it is brittle and easy to crack. The elastic modulus of polymer material is close to bone tissue, but its strength and surface osteogenic activity remain to be further studied. Titanium and its alloys have good biocompatibility, corrosion resistance, and mechanical properties, including hardness, tensile strength, yield strength, and fatigue strength, which meet the functional requirements of oral implants [17,18,19]. As implant materials, titanium and its alloys have been widely used in clinical practice and have achieved good repair effects [20,21,22].

**Figure 1:**
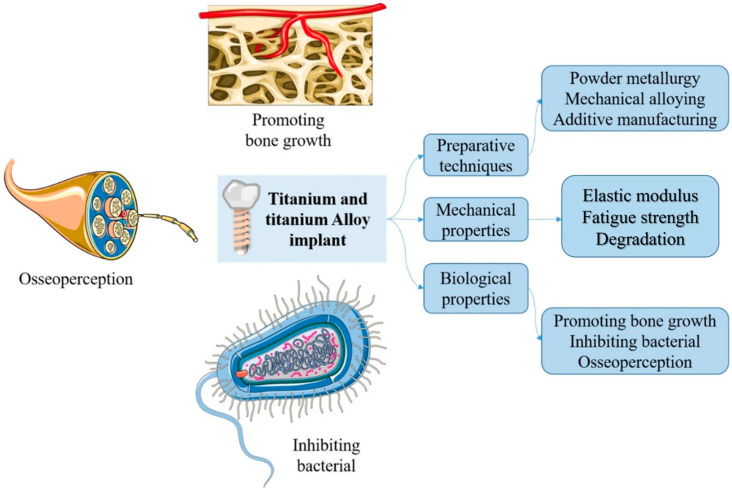
Preparative techniques, and mechanical and biological properties of titanium and its alloy implant.

This study provides a comprehensive compendium regarding the techniques used to manufacture titanium and its alloy oral implants in the last 5 years. Properties like mechanical properties and biological properties have also been concluded. We believe that this review would be able to and, therefore, stimulate their future development and biomedical applications (Figure 1).

## 2. Materials and Methods

The present work reviewed titanium and its alloy oral implants by Demiris et al.’s search strategy [23]. The following electronic databases were primarily searched to identify relevant studies: Science Direct, Google Scholar, and PubMed, using the terms “preparation” OR “properties” OR “Powder metallurgy” OR “Mechanical alloying” OR “Additive manufacturing” OR “Degradation” OR “Coating” OR “TiO_2_ nanotubes” OR “PLGA Coating” OR “Chitosan Coating” OR “Osseointegration” OR “Antibacterial” OR “Osseoperception” together with (AND) “titanium implant” OR “Ti implant”. Furthermore, the bibliographies of selected suitable articles and previously published reviews dealing with the biological complications of implants were searched to identify other potentially relevant material. We included pertinent in vivo and in vitro experiments. Two authors independently evaluated the titles and abstracts of the selected articles followed by a thorough joint discussion of the authors to establish and resolve any disagreement between them concerning the articles.

## 3. Techniques Used to Manufacture Titanium and Its Alloy Dental Implants

**Figure 2:**
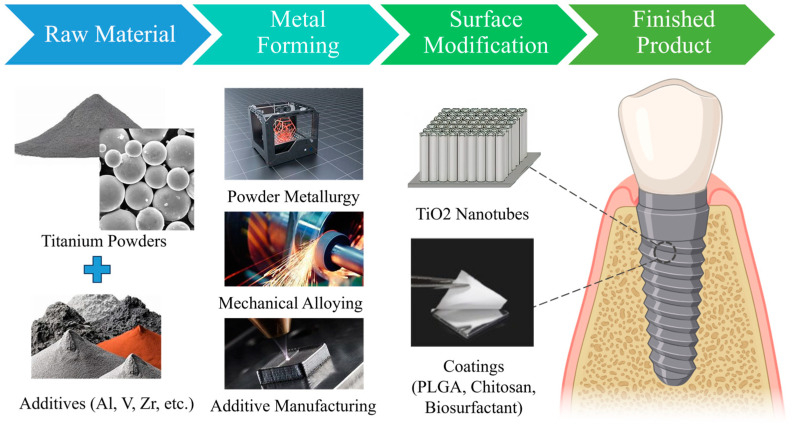
Scheme demonstrating the processes of producing dental implants.

Numerous techniques have been used to fabricate titanium and its alloys in dental implants. Generally, powder metallurgy, mechanical alloying, and additive manufacturing are the three standard methods for titanium implant fabrication [24]. Figure 2 presents a concise overview of processes used in producing dental implants, while this section delves into the advantages, influencing factors, and principles associated with these techniques, as outlined in Table 1.

### 3.1. Powder Metallurgy

Powder metallurgy (PM) is a technique that makes powders or uses metal powder (or metal powder and non-metal powder mixture) as raw materials to manufacture metal materials, composite materials, and various types of products after forming and sintering [25]. Powder metallurgy is similar to ceramic production; both belong to powder sintering technology. Therefore, new powder metallurgy technologies can also prepare ceramic materials. Materials manufactured by powder metallurgy have remarkable mechanical and physical properties [26] which traditional casting methods cannot obtain. Porous, semi-dense, or completely dense materials can effectively convey through powder metallurgy. Because of the benefits of powder metallurgy innovation, it has become a way to tackle the issue of new materials and assumes a conclusive part in growing new materials [27]. Powder metallurgy has the following advantages:

1. It can minimise the segregation of alloy composition and dispose of the coarse and uneven casting texture.
2. It can handle the composite of many materials, gives full play to the particular qualities of every part material, and is a minimal expense creation of superior perform metal matrix and ceramic composite method.
3. It can produce materials and items with exceptional design and properties that cannot be created by conventional smelting strategies. For example, new porous biomaterials, porous division membrane materials, superior performance structural ceramic abrading apparatuses, utilitarian ceramic materials, and so on.
4. It can lessen the creation’s asset and energy utilisation through close net arrangement and programmed bunch creation.

### 3.2. Mechanical Alloying

Mechanical alloying (MA) refers to a powder preparation technology in which metal or alloy powder is repeatedly cold welded and fractured through long and intense impact and collision between powder particles and grinding balls in high-energy ball mills, resulting in atomic diffusion in powder particles to obtain alloyed powder [32,33,34]. Mechanical alloying can avoid the high-temperature melting and solidification process of standard metallurgical methods, achieve alloying at room temperature, and obtain uniform alloy with fine structure and high yield.

Mechanical alloying powder is unlike the alloy material formed after metal or alloy casting, which is fully interatomic bonding between components to create a uniform solid solution or compound [35]. In most cases, only the elements are allowed to reach or approach atomic distances at the points, lines and planes in contact during the limited ball-milling time, and the final result is a mixture or compound with a very uniform distribution of the elements. When the ball grinding time is very long, alloys or compounds can be formed in some systems through solid-state diffusion to make the components reach interatomic bonding.

In the early stage of ball milling, we repeated extrusion deformation after crushing, welding, extrusion, and laminated composite particles. Under the continuous action of the ball mill mechanical force, the composite particles produce a new atomic surface, and the layered structure is refined continuously. In the mechanical alloying process, the formation of lamellar structure marks the beginning of interelement alloying. The reduction of lamellar spacing shortens the diffusion path between solid atoms and accelerates the interelement alloying process. During ball milling, the harder the powder is, the more complex the recovery process is, and the smaller the grain size that can be achieved by ball milling. In addition, the higher the hardness of the material is, the higher the dislocation slip is to carry out, and the dislocation density in the lattice is higher, which provides a fast diffusion channel for the alloying process [40], which further speeds up the alloying process. During the time spent ball milling, an enormous number of crashes happen between ball and powder ball, and the captured powder deforms seriously under the impact, making the powder subjected to the “miniature” forging action of the two collision balls. The high-density defects and nano interface created by ball milling greatly promote the self–propagation high–temperature synthesis (SHS) response and assume the leading part. After the reaction, the mechanical ball milling was continued, and the cold weld-breaking-cold welding process of the powder was forced repeatedly to refine the powder and obtain nanocrystals. Mechanical alloying is a complex process, so a series of influencing parameters must be optimised to obtain the ideal phase and microstructure [32,36].

### 3.3. Additive Manufacturing

Additive manufacturing (AM), ordinarily known as 3D printing, is a combination of computer-aided design (CAD), processing materials, and moulding technology on account of computerised model files through programming and mathematical control framework, specialised metal materials, non-metal materials and medical-biological materials, as per the extrusion, sintering, melting, light curing, spraying and other ways of stacking, and manufacturing technology to produce solid objects [38]. In contrast with the customary handling method of raw material removal, cutting and assembly, it is a “bottom-up” production technique through the material accumulation without any preparation [38]. This method makes it conceivable to produce complex underlying parts confined by customary assembling techniques before [39].

AM technology has improved quickly in the past two decades [41]. Various names, for example, “Rapid Prototyping”, “3D Printing”, and “Solid free-form Fabrication”, separately express the qualities of this innovation from various sides. Additive manufacturing technology alludes to a logical and mechanical framework that straightforwardly fabricates parts driven by 3D data based on the discrete-stacking guideline. AM technology does not need customary tools and jigs and a multi-channel handling process. One piece of equipment can rapidly and precisely produce arbitrary complex shape parts, accordingly understanding the parts “free manufacturing”, tackle the framing of numerous mind-boggling structure parts [42], and extraordinarily decrease the processing procedure, abbreviating the processing cycle. Furthermore, the more complicated the item structure, the more critical the impact of manufacturing speed. To obtain additive manufacturing materials with ideal properties, the following aspects are required to control:

1. The first is the control technique of the material unit. Controlling the physical and chemical changes of material units during the time spent stacking is troublesome. For instance, in direct metal forming (DFM), the little molten pool’s size and the outside air control straightforwardly influence the manufacturing exactness and the presentation of the parts.
2. The second is the recoating technology of hardware. Automatic coating in additive manufacturing is an essential course of material accumulation, and the procedure of recoating straightforwardly decides the accuracy and quality of parts in the direction of accumulation. The layer thickness is created towards 0.01 mm. Controlling the same layer thickness and stability is essential to improve accuracy and decrease the surface roughness.
3. The third is manufacturing technology efficiency by increasing material fabrication to the oversize components manufacturing technology development. For instance, it takes too long to manufacture alloy structural parts on aircraft directly by metal laser. Therefore, it is not easy to realise the synchronous manufacturing of multiple laser beams, improve the manufacturing efficiency, ensure the consistency between simultaneous additive associations, and combine the regional quality of manufacturing.

The traditional preparation process has great advantages in the manufacture of implants. Powder metallurgy, mechanical alloying, and 3D printing are three traditional methods for preparing implants. Implant materials with good properties can be obtained by adjusting preparation parameters and selecting suitable preparation methods according to materials. With the development of science and technology, it is of extraordinary importance to have innovation in new preparation techniques to improve the performance of implants, such as mechanical compatibility. In addition, it is also very important to change the stress distribution, hardness, and strength of implants with appropriate post-treatment methods. In the future, various preparation methods can be used to optimise implants and prepare implants with specific adaptations according to the characteristics of tissues.

## 4. Properties of Titanium and Its Alloy Oral Implant Material

Implants need to meet specific properties to improve implant stability and long-term survival. This section will discuss the implant’s mechanical and biological properties (Table 2).

### 4.1. Mechanical Properties

The biomechanical properties of dental implants have attracted more and more attention [44,45] because there are a series of mechanical problems in the selection of materials, implant design, and clinical application. Firstly, the implant should withstand functional loads and have sufficient strength to ensure no serious deformation or fracture occurs. Secondly, the implant should transfer enough stress to the surrounding bone tissue to avoid wasteful bone atrophy. Finally, the stress transfer of the implant to the surrounding bone tissue should not exceed physiological limits to prevent bone resorption and fracture. In this part, we reviewed the mechanical properties of implants, such as hardness, tensile strength, yield strength, fatigue strength, etc.

#### 4.1.1. Elastic Modulus

**Figure 3:**
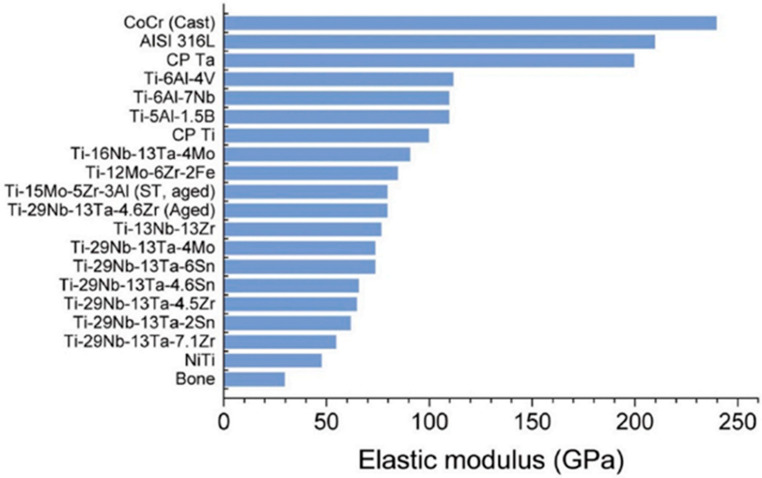
Titanium and its alloy’s elastic modulus compared with bone, 316L stainless steel, and Cobalt-chromium alloys [124]. Figure reprinted with permission from CC BY-NC-ND 4.0.

Implants with good biomechanical properties play an essential role in the long-term success rate of implants [46]. After implantation, the implant forms a bone-binding interface with the surrounding bone tissue [47]. When the implant is under load, the stress generated by the implant-bone interface includes compressive stress, tensile stress, and shear force. Tensile stress and shear force can cause a fracture tendency of the bone interface. Studies [123] have shown that the compressive strength of the surrounding bone is much stronger than the tensile strength and shear strength. Therefore, it is necessary to improve the compressive stress of bone tissue and transfer it to the surrounding bone tissue for the long-term success rate of implantation. The elastic modulus is the proportion of stress to strain in the elastic stage of the material. An appropriate elastic modulus can give the dental implant good biomechanical properties [48,49]. A proper elastic modulus is beneficial for transferring stress at the junction surface between implant and bone to the surrounding bone and avoiding the “stress shielding” effect, resulting in bone atrophy and implant displacement. The elastic modulus of existing commercial titanium implants is about 110 GPa [50]. In comparison, human cortical bone is about 12–18 GPa [47], and cancellous bone is about 0.1–4.5 GPa [47], leading to the mismatch between the elastic modulus of implants and human bone tissue. Therefore, titanium implants with porous structures have been developed to obtain implants that match the elastic modulus of bone tissue, avoid stress concentration and stress barrier when implants are under load, and improve the survival rate of implants [52,53]. Ou, et al. [54] prepared cytocompatibility Ti-xZr alloys as dental implant materials. The outcomes showed that the alloy exhibited low elastic modulus and high mechanical strength, contrasting with pure mercantile titanium. In addition, the Ti-xZr alloy possessed the optimum strength/elastic modulus ratio and osteogenic activity. Figure 3 demonstrates titanium and its alloy’s elastic modulus compared with bone, 316L stainless steel, and Cobalt-chromium alloys.

#### 4.1.2. Fatigue Strength

Fatigue refers to the change in the properties of a metal material under sudden stress or strain. Fatigue life refers to the number of cycles or time required for a material or part to reach fatigue failure under sudden change stress. The stress level of the material or part, ability to resist damage, structure, and surface morphology will affect fatigue life [55,56]. Dental implants must bear the stress caused by chewing countless times in the oral cavity. Coupled with the special oral environment, the implant will experience fatigue and fracture which affects the service life of the implant. There are many reports of implant and central screw loosening and even breaking caused by mechanical fatigue [57,58]. Therefore, it is necessary to study the fatigue properties of implants. Examinations have shown that the main factors affecting the fatigue failure of the implant were the strength of the implant material, implant size, and structural design. The fatigue strength of an implant is mainly related to its length, diameter, shape, and structure. Implants with a diameter greater than 4.5 mm were considered large-diameter implants, and those with a diameter less than 3.5 mm were considered small [59]. Large-diameter implants can increase the area of bone and implant interaction [69], improve the stress distribution of the implant-bone system, increase the fatigue limit of the implant, and increase the service time of the implant. Implant length also has an impact on its survival time. Short implants are generally believed to have a higher probability of short-term failure, and the failure rate of implants shorter than 7 mm increases significantly. In general, increasing the screw density of the implant (decreasing the pitch) can increase the stress area of the implant, but too many threads can also lead to fatigue damage at the bone interface. Studies have shown that the optimal pitch for cylindrical threaded implants is between 0.8–1.2 mm [70]. Sun, et al. [57] studied the impact of loading angles and implant lengths on dental implants’ static and fatigue fractures. The outcomes showed that 9 mm length implants have a higher static failure load and can withstand greater bending moments, while 11 mm length implants have a more extended fatigue life. Likewise, as the loading angle increases, the static strength and bending moment decrease linearly, and the fatigue life shows an exponential decrease three times.

**Figure 4:**
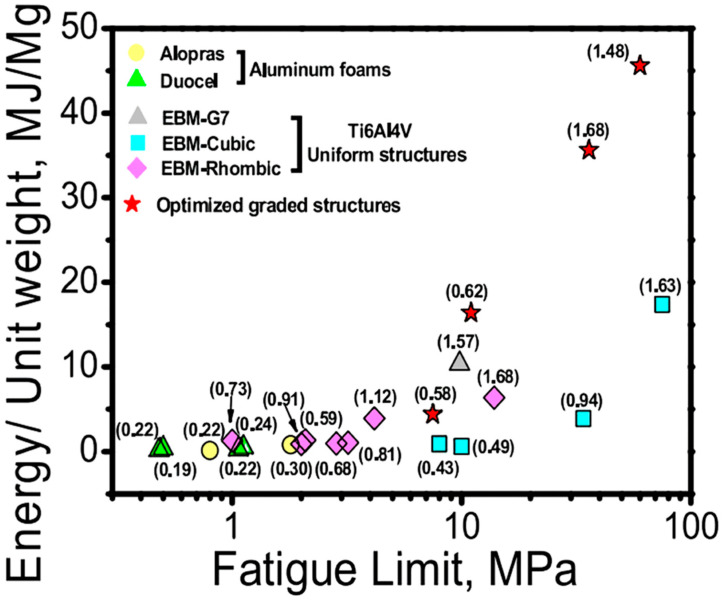
Comparison of the fatigue strength and the absorption of the graded meshes with the uniform reticulated EBM Ti-6Al-4V meshes and metallic cellular structures. The numbers in parentheses indicate the densities of the cellular structures [125]. Figure reprinted and adapted with permission from Elsevier.

The fatigue properties of implants are closely relevant to the comprehensive mechanical properties of implants. The stronger the material, the better the plasticity, the better the fatigue performance, and the longer the service life of the implant. Therefore, using certain strengthening methods to enhance the strength of pure titanium implants is also conducive to further improving the fatigue performance of pure titanium implants. Figueiredo, et al. [71] used equal channel angular extrusion prepared pure titanium, and its strength, elongation, and other mechanical properties were studied. In addition, the fatigue properties of treated titanium implants were studied. The results showed that the strength of pure titanium is increased from 400 MPa to 780 MPa by equal channel extrusion. Elongation dropped from 20% to about 8%. The fatigue strength increased from 220 N to 250 N. Ren, et al. [125] reviewed the fatigue behaviour of Ti-6Al-4V cellular structures fabricated by additive manufacturing technique. Figure 4 shows the fatigue durability and absorption characteristics of the graded mesh configurations in comparison with the uniform reticulated EBM Ti-6Al-4V meshes and metallic cellular structures.

Implants are built based on the development of modern mechanical processing [126]. Good processing performance can meet the needs of morphological design, and occlusion is the basic function of teeth [127]. Implants can bear static and dynamic chewing force to avoid fracture, deformation, and wear in the long-use process. In addition, an implant with good mechanical properties is more similar to the modulus of bone tissue, which can avoid excessive stress concentration between bone tissues when the implant is stressed [128]. The implant’s structure and surface treatment will affect the bone binding rate and long-term stability after implantation. Implants with good mechanical properties can adapt to the speed of bone bonding to achieve long-term stability. In addition, the implant with high hardness and strength has a fast planting speed and long service life [129]. Implants with strong retention and stability can effectively restore the chewing function of teeth. After implantation, the tooth feels natural and more comfortable in the mouth than traditional therapeutic techniques.

#### 4.1.3. Degradation

Titanium and its alloys were once considered ideal implant materials due to their relatively low density, robust strength, and high resistance to fatigue and corrosion [63]. Their modulus of elasticity is closer to bone than any other implant material, except for pure titanium [64]. However, recent research suggests that titanium particles as a product of degradation have been detected in peri-implant oral tissues, and it has been assumed that implants were the source of these particles [66]. Suárez-López del Amo et al. [61] systematically reviewed that titanium particles are commonly found surrounding peri-implant tissues. For these reasons, the effects of Ti and its alloy degradation are worth discussing:

- Mechanical wear-related degradation: The dental implant surface may experience mechanical abrasion in various stages of its lifecycle, including the initial implantation surgery, the subsequent attachment of implant-supported prostheses, routine mechanical cleaning to prevent issues and therapeutic interventions for peri-implant infections. It can also be subject to friction due to the micro-movements between the implant and its supra-structure when the implant is in use. Deppe, et al. [67] used in vitro research to show that the placement of dental implants can induce the liberation of particles that become detached from their surface during the implant’s insertion into the bone. This finding is confirmed by Suárez-López del Amo et al. [61]. Their review revealed the presence of titanium debris in the crestal region of the osteotomy site, characterized by round, elongated, or small angular particles.
- Chemical agent-related degradation: Some chemical agents will decrease the protection provided by the oxide layer forming on the surface of dental implants, causing a corrosion process [60]. Kotsakis, et al. [130] demonstrated that there was a reduction in the defensive characteristics of the oxide layer upon the inclusion of fluoride ions.
- Bacterial biofilm-related degradation: Evidence shows that the oral cavity usually exhibits an acidic environment. Surrounded by low pH, saliva favours the exchange of ions between saliva and the implant surface, which triggers higher corrosion rates [68]. Safioti, et al. [62] analysed specimens obtained from 30 patients, examining the presence of dissolved titanium within the submucosal plaque. They observed a substantially higher bacterial load and a pronounced elevation in titanium ion concentrations in the samples from sites afflicted with disease compared to the specimens from healthy sites. In general, the research findings indicate that an acidic environment promotes the mechanisms that trigger the release of titanium particles, and it is highly inclined to stimulate the peri-implantitis processes associated with bacterial biofilm formation [65].

### 4.2. Biological Properties

#### 4.2.1. Promoting Bone Growth

**Figure 5:**
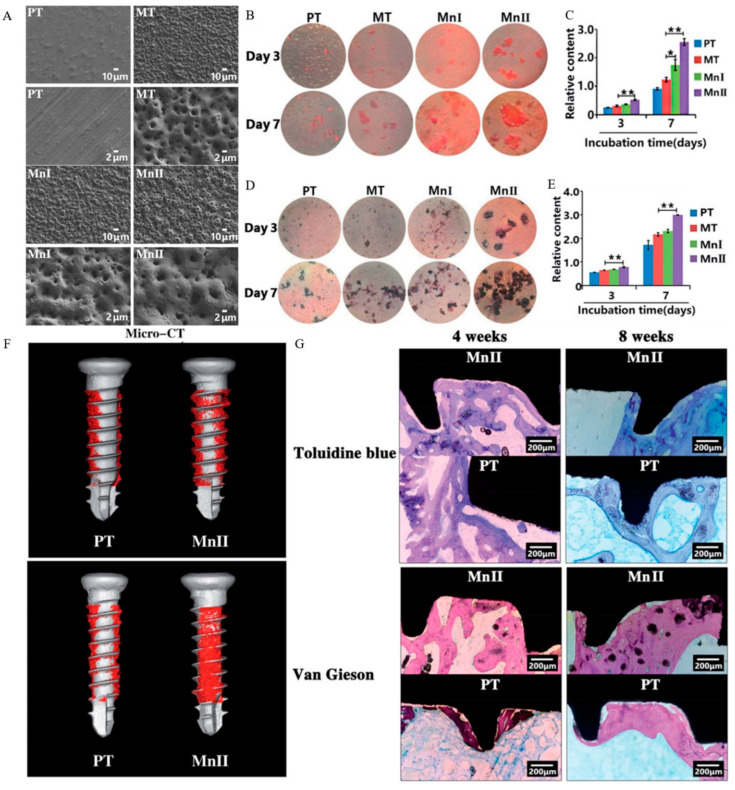
(**A**) SEM images of pure titanium, MAO titanium, manganese-titanium dioxide I, and manganese-titanium dioxide II under low and high magnification. (**B**) Culturing for 3 and 7 days. ECM mineralised nodules generated by cells cultured on different samples with (**C**) ECM relative content. * *p* < 0.05 and ** *p* < 0.01 compared to MT. (**D**) After 3 and 7 days of culturing, ALP was generated by cells cultured on different samples and results with (**E**) ALP relative content. ** *p* < 0.01 compared to MT. (**F**) Micro-CT images of the bone-implant junction surface at 4 and 8 weeks post-implantation. (**G**) Toluidine blue and van Gieson images of the bone-implant interface at 4 and 8 weeks post-implantation. (**A**–**G**) Figures reprinted and adapted with permission from Taylor & Francis [86].

Titanium and its alloys are predominant materials for dental implants due to their steady chemical properties and outstanding biocompatibility [20,72]. Be that as it may, aseptic loss may lead to implant removal. The tiny gaps mainly bring about aseptic loosening in prosthesis bone connection points [73]. Under gravity and pressure, the tiny gap between the implant and the wear particles will amass at the interface and impede the direct contact between the implant and bone. Together with local inflammation and cascade reactions activated by immune cells, the osteoblast was enriched, and bone resorption occurred, which occasioned implant loss [74]. To improve the stability of the implant and avoid implant loosening, many scholars have modified the surface of the implants from the physical and chemical perspectives to construct a coating with bone conduction or bone induction function to improve the bone integration rate of implants and thus, enhancing the stability of implants [75,76,77]. Examinations have found that changing the implant’s surface roughness and providing a porous structure can bring more adhesion sites for cells which benefits cell adhesion [78,79]. Therefore, changing the surface roughness of materials is an effective method to improve the bone integration rate. Giving a porous structure to the surface of a material is one way to change the roughness. Micro arc oxidation (MAO) [80,81], dealloying [82,83], and 3D printing [84,85] are three main methods of preparing porous coatings. Zhao et al. prepared [86] manganese-titanium dioxide (Mn-TiO_2_) microporous coating by MAO on a titanium surface. The results indicated that the Mn-TiO_2_ microporous biotic coating can promote the adhesion, proliferation, differentiation, and mineralisation of MC3T3-E1 osteoblasts. In addition, in vivo experiments indicated that the coating can promote early osseointegration (Figure 5). Coatings rich in biologically active ions were developed further to improve the bone integration ability of the materials. Ke, et al. [87] prepared slant hydroxyapatite (HA) coating on Ti6Al4V by Laser Engineered Net Shaping (LENS^TM^) followed by plasma spray deposition. The results indicated the existence of an interfacial layer by LENS^TM^-reinforced adhesive bone strength.

Alkali heat treatment [88,89] and electrochemical anodisation (EA) [90,91] can prepare nanostructures such as nanoparticles, nanorods, and nanotubes. Studies have found that nanoparticles have more benefit to osteogenesis due to the nearer specific surface area. Zeng, et al. [92] treated titanium with alkali to make its surface nanostructured. The results indicated that alkali-treated titanium had a higher bovine serum albumin and human fibronectin absorbance ability and enhanced apatite formation. In vivo, results showed that titanium with surface nanostructures can promote osteointegration.

From the perspective of improving the biological properties of materials, enhancing the biocompatibility of titanium and its alloy surface, and promoting the osseous energy are also beneficial to improving the osseous integration ability of materials. Coating natural biological materials (such as biological proteins, growth factors, etc.) on the surface of titanium and its alloys can significantly improve the surface biocompatibility of materials [93]. Because these natural biomaterials have natural cell-recognition sites on their surfaces, they are more conducive to cell-material interaction. From the perspective of promoting surface osteogenesis, bone morphogenetic proteins (BMPS) are a group of highly conserving functional proteins with similar structures that stimulate DNA synthesis and cell replication, thereby promoting the directed differentiation of mesenchymal cells into osteoblasts and inducing bone and cartilage formation [94,95]. Zhu, et al. [96] prepared porous titanium alloys using the electron beam melting (EBM) technique with a temperature-sensitive collagen blend with recombinant human BMP9 as a composite skeleton regularly provided to enhance osteogenesis. The results indicated that the composite scaffold has good biocompatibility and provided bioactive growth factors for bone repair. In addition, the release of BMP9 strengthened osteogenesis around the skeletons.

**Figure 6:**
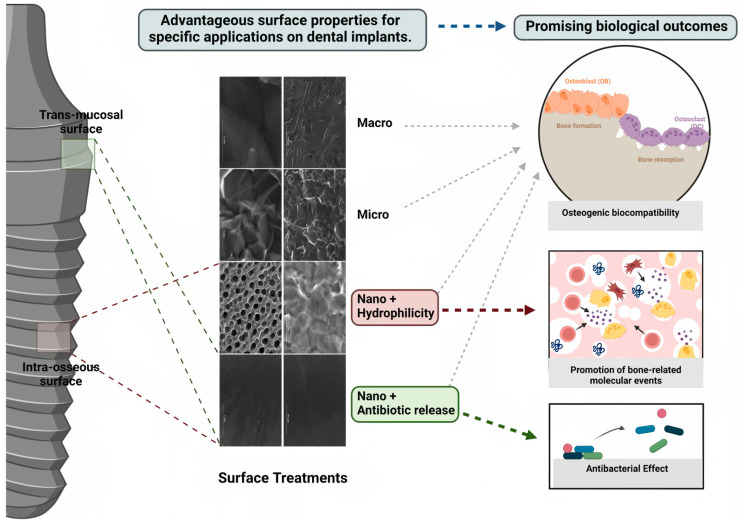
Schematic illustration demonstrating the major promising biological outcomes of each surface topography. Figure reprinted with permission from Creative Commons Attributes (CC BY) [132].

It is worth mentioning that surface treatment could increasingly promote bone growth. Schwartz et al. [131] prepared bioactive coatings on Ti-6Al-4V alloy by Plasma Electrolytic Oxidation (PEO). Coating microstructures prepared via microarc oxidation in molten salts allows osteoblasts to attach to the implant surface more effectively using structural proteins and glycoproteins. Kunrath et al. [132]. prepared macro-, micro-, and nano-topographies on dental implant surfaces. The results showed that hydrophilic nanotextured surfaces promote favourable properties to stimulate early bone-related cell responses, favouring their application in bone-anchored surfaces. Furthermore, the researchers delved deeper into antibacterial properties, especially in biomaterials with drug-delivery systems (BDDS) and nanofeatures [133]. The following chapter will detail antibacterial properties (Figure 6).

#### 4.2.2. Inhibiting Bacteria

In addition to bone integration, peri-implant mucositis [97] and other infections can also affect the success of implants [98]. Studies have shown that about 29.48% (implant-based) and 46.83% (subject-based) of dental implants endure peri-implant mucositis, and about 9.25% (implant-based) and 19.83% (subject-based) evolve into peri-implantitis. Peri-implant mucositis is a biofilm-induced inflammation that grows on the soft peri-implant mucosa. It develops from healthy peri-implant mucosa around osseointegrated dental implants after accumulating bacterial biofilms [99]. To solve this problem, antimicrobial coatings have been developed to restrain and forestall bacterial infections [100,101,102]. The titanium surface must be treated by adding material or agents in coatings to forestall bacterial colonisation. This section will discuss some strategies for endowing titanium surfaces with antimicrobial properties.

Surface coatings with bacteria-inhibiting [103] or bacteria-killing [104] materials are the two main methods of imbuing titanium and its alloys with antimicrobial properties [105]. Bacteriostasis means repelling bacteria without killing them and keeping them away from the implant’s surface. Sterilisation refers to the destruction of the bacterial membrane or cell wall, infiltration of a cell wall, destruction of internal DNA to hinder bacterial proliferation, production of reactive oxygen species, blocking ATP synthase and stopping cell respiration from eliminating bacteria, and further restriction of biofilm formation.

Traditional antibacterial materials include polyethene glycol [106], plant polyphenol [107], and biosurfactants [108]. Nowadays, nanotextured surfaces have become a relatively new field in bacteria in this complex oral environment, and they may support longstanding peri-implant health [134]. Typical coatings are demonstrated in the following paragraphs:

- TiO_2_ nanotubes: Gunputh, et al. [135] established coating TiO_2_ nanotubes (NTs) with silver nanoparticles (Ag NP) on titanium implants. Evidence showed that TiO_2_ NT-Ag NP possesses outstanding antibacterial properties. Turu, et al. stepped further on TiO_2_ NTs with Ag NP and reduced graphene oxide (rGO) on Ti6Al4V-ELI. Results showed that TiO_2_ NT-Ag NP has a reducing effect on the attachment of *S. aureus*. TiO_2_ NT-rGO had a retarding effect on the maturation process of the biofilm [136]. Zhang, et al., through hydrothermal treatment, incorporated Graphene quantum dots (GQDs) into TiO_2_ nanorods (TiO_2_ NR) on the Ti implant surface, finding that GQDs-modified TiO_2_ NR on a Ti implant can improve the antibacterial activity [137].
- Poly(lactic-co-glycolic acid) (PLGA) coating: Kazek-Kęsik, et al. [138] loaded amoxicillin in a PLGA layer on Ti-15Mo alloy. Turnout coating released drugs can inhibit the growth of *S. aureus* and *S. epidermidis*. Noothongkaew, et al. [139] loaded ZrO_2_ NTs in PLGA dip-coating steps. Results showed that PLGA with ZrO_2_ NTs exhibited mild self-antibacterial properties. Zhao, et al. [140] functionalized PLGA containing sodium dodecyl sulfate (SDS) and levulinic acid (LA). PLGA−SDS/LA coating exhibited antibacterial properties against *S. aureus* and *E. coli* were 98.9% and 99.8%, respectively.
- Chitosan (Chi) coating: Chen, et al. [141] synthesized Chi/cefazolin coatings by electrophoretic deposition on Ti and alkali-treated Ti surfaces. It was found that fabricated Chi/cefazolin coatings exhibited high antibacterial activities against *E. coli*. Andrade del Olmo, et al. [142] created Chi-based genipin (Ti-CHIGP) and polyethene glycol (Ti-CHIPEG) hydrogel coatings on the Ti6Al4V surface. Results showed an effective bifunctional antibacterial activity (bacteria-repelling + contact killing) against *S. aureus* and *E. coli*. Bose, et al. [143] added Cu_2_O@CuFeS_2_ into the Chi-HA matrix. It was found that the Cu_2_O@CuFeS_2_ composite exhibited a high antibacterial efficacy against *S. aureus* (~98.5%) and *E. coli* (~96%) under NIR light irradiation stimuli.
- Biosurfactant: Tambone, et al. [109] prepared a rhamnolipid biosurfactant R89 (R89BS) coating on titanium implants to reduce microbial biofilm formation. The results indicated that R89BS significantly inhibited the formation of a microbial biofilm, and the biofilm metabolic activity was reduced.

Other bacteria-inhibiting methods are sterilization where bactericidal materials are coated on the titanium alloy surface or compounded into the titanium alloy for a long-term release of bactericidal effects. Silver [110], copper [111], zinc [112], chlorhexidine [113], and some antibiotics [114,115] are promising antimicrobial materials. Zhou, et al. [116] prepared an F-doped TiO_2_ microporous coating on titanium to enhance bacterial resistance. The results indicated that a high F addition could significantly improve bacterial resistance and osteogenesis. Massa, et al. [117] synthesised a novel antibacterial composite coating for titanium due to highly ordered nanoporous silica and silver nanoparticles. The results indicated that the coating produced a forceful antibacterial effect on the titanium surface by killing the adherent bacteria and reducing the scope of biofilm formation.

**Figure 7:**
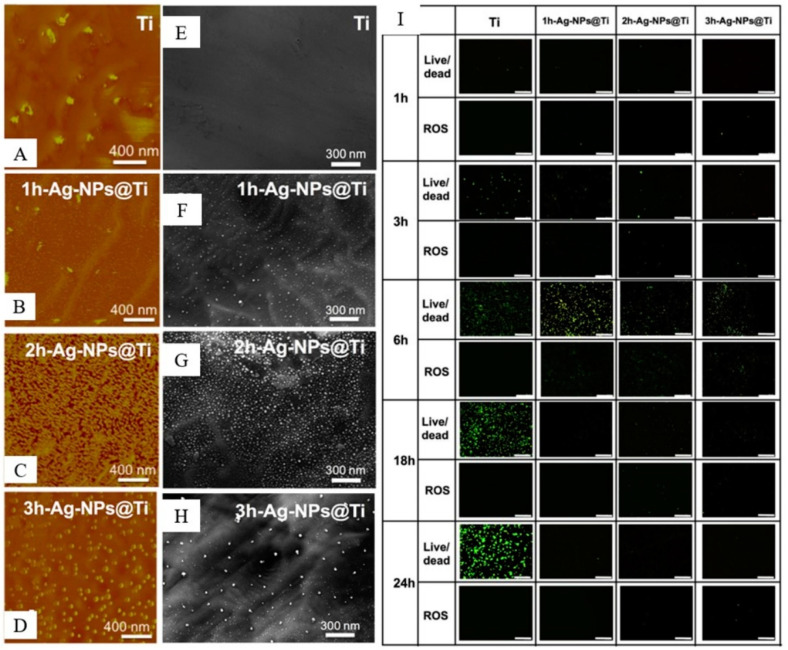
Sample characteristics: AFM (**A**–**D**) and SEM (**E**–**H**) observed surface morphology, corresponding to 1 h, 2 h and 3 h, respectively. (**I**) Different cultivation time points live/dead staining and intracellular ROS staining results. (**A**–**I**) Figures reprinted and adapted with permission from Elsevier [119].

Studies have shown that the size of the material may affect the antibacterial effect. *S. aureus* size is about 500–800 nm. Peng, et al. [118] found that a larger surface is more conducive to inhibiting bacterial growth. A similar result was found in the research of Wang, et al. [119]. The results indicated that the Ag nanoparticle size would influence the antibacterial effect (Figure 7). Likewise, Cao, et al. [120] discovered that the antibacterial activity of smaller Ag nanoparticles was less than the larger ones because of the larger land area, which was like the tracking down in this study that the antibacterial performance of larger globule Ti_2_Cu and bulk β phases was better than the small ones.

#### 4.2.3. Osseoperception

**Figure 8:**
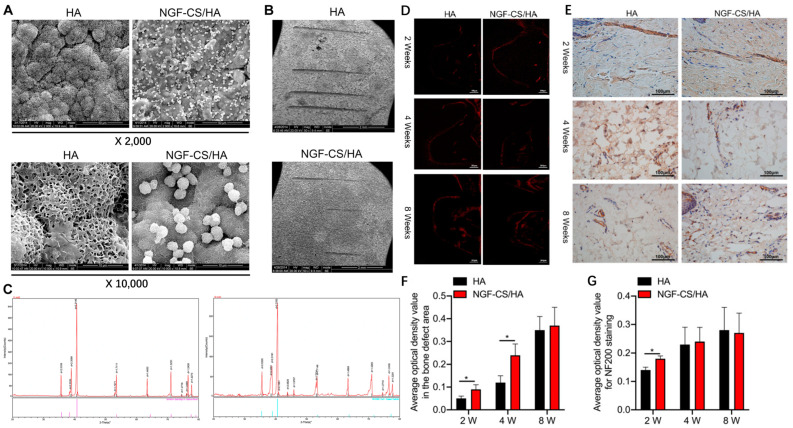
(**A**) SEM images of surface topography of HA, NGF-CS/HA coatings. (**B**) Scratch in NGF-CS/HA composite coating SEM images. (**C**) XRD spectrums of HA and NGF-CS/HA coatings. (**D**) The number of nerve fibres at 2, 4 and 8 weeks in HA- and NGF-CS/HA-coating implant. (**E**) The expression of NF200 at 2, 4 and 8 weeks in HA- and NGF-CS/HA-coating implant. Quantifying the average optical density value in the defect area (**F**) and NF200 staining (**G**). HA, hydroxyapatite; NGF-CS/HA-coating, nerve growth factor-chondroitin sulphate/hydroxyapatite-coating. * *p* < 0.05. (**A**–**G**) Figures reprinted with permission from Creative Commons Attributes (CC BY) [144].

The implantable denture has been used in clinics because of its advantages of good retention and stability, avoiding damage to adjacent teeth, and good aesthetics. Compared with natural teeth, implants lack a periodontal membrane and its receptors. A study found that patients with implants could also sense the implant in the mouth and confirm functional movement of the mouth through implants lacking periodontal proprioceptors [121]. Some studies have found that this may be related to nerve fibres around the implant which regenerate from the remaining nerve fibres around the implant and make the implant responsive to external mechanical loads [122]. Notwithstanding, because of the low density of these nerve fibres, the sensory threshold of implants is 10–100 times that of natural teeth. Patients with implants may be unable to perceive a large chewing force over time, which may lead to a long-term overload of implants. These adverse factors can easily cause implant loosening, surrounding bone absorption, and even damage to implant dentures resulting in implant failure. Thus, it is of great clinical significance to promote the regeneration of nerve fibres around implants and improve the chewing sensation of patients with implants for the long-term effects of implants. It is an effective method to promote nerve regeneration around implants by coating it with materials that can stimulate nerve regeneration on the surface of implants. Ye, et al. [144] prepared a nerve growth factor-chondroitin sulphate/hydroxyapatite-coating (NGF-CS/HA-coating) composite implant to induce nerve regeneration. The results indicated that the implantation of NGF-CS/HA-coating implants elevated the morphology of nerve fibre in the mandible of Beagle dogs. In addition, the NGF-CS/HA-coating implants also upregulated the levels of neurogenic differentiation-related genes (Figure 8). Examinations have discovered that the topological structure can affect a series of behaviours of cells and forward a variety of tissue repair and regeneration processes [145,146,147,148]. Tamplenizza, et al. [147] worked out the differentiation of PC12 cells on nanostructured TiO_2_ films by supersonic cluster beam deposition. The results indicated that the roughness of nanostructured TiO_2_ provoked neurogenesis by activating the expression of nitric oxide synthase and the photo-extracellular signal-regulated kinase 1/2 signalling.

Nowadays, the majority of implants are made of titanium and its alloys. The research surface of titanium metal has good biocompatibility, high mechanical strength, stable chemical properties, and can form good bone bonding with bone tissue. Nevertheless, titanium, as an inert metal, cannot stimulate osteoblast and osteocyte proliferation and depends on mechanical locking with alveolar bone to provide retention force. Therefore, it enhances the bond between implant and bone, preventing bone tissue absorption around the implant and providing long-term stability. Surface coating technology of the implant surface through certain technical means to increase the surface’s physical and chemical properties, using the change of implant and bone tissue after implantation of the direct contact surface can promote the implant and bone tissue surface faster, resulting in better bone union or a planting body giving better bioactive functions such as antibacterial, promotion of bone perception, etc.

## 5. Conclusions and Perspectives

Titanium and its alloy implants have many remarkable properties such as biocompatibility and corrosion resistance, as well as good mechanical properties, including hardness, yield strength, tensile strength, and fatigue strength. To improve the stability and long-term survival rate of implants, many physical, chemical, and biological modification studies have been performed on implants to improve the ability of bone integration and antibacterial and peripheral nerve regeneration. However, there are still many issues concerning the part of titanium and its alloy implants as reformative biomedical materials are ignored and uncharted. Many endeavours are needed to examine the different behaviours of cells and their synergy with titanium and its alloy implant. Essential but uncharted problems can be classified into the following aspects:

1. Even though many methods have been developed and examined to change the mechanical properties of titanium and its alloy implants, the mechanical properties are still imperfect compared to natural bone tissue. The current strategy is to change the mechanical properties of the implant by combining different metal elements or changing the structure of the implant. More advanced processing techniques need to be explored to adjust the mechanical properties of titanium and its alloy implant.
2. Existing studies usually prepare bone-promoting and antibacterial coating on implant surfaces to increase bone integration and antibacterial ability. However, how to ensure the balance between bone integration and antibacterial, and whether antibacterial drugs, biomolecules or materials will affect bone integration still needs further research.
3. Changing the topological structure of the implant surface can affect cellular behaviour and thus promote osseointegration, but the related mechanisms must be further verified. Whether the surface topological structure will impact the mechanical properties of implants needs to be further studied.
4. Promoting neuralisation and vascularisation of the tissues around implants is conducive to improving the microenvironment of the surrounding tissues. It is more conducive to enhancing the bone integration ability of implants. Future studies can combine nerve and vascular regeneration to promote bone integration of implants to improve its stability.
5. To avoid drug resistance and improve antibacterial effects, implants that can control the release of antibacterial drugs or intelligently respond to drug release can be prepared according to the timeliness of inflammatory reactions in the future to achieve better antibacterial effects.

## Figures and Tables

**Table 1:** Techniques used to fabricate titanium and its alloy implants.

Methods	Remarks on Fabrication Methods	References
Powder metallurgy	Limits the segregation of alloy composition and erases the coarse and uneven casting structure.	[20,21,22,25,26,27,28]
	Prepares a progression of high-performance non-equilibrium materials.	
	Produces materials and products with unique structures and properties.	
	Implementation of near-net formation and automatic batch production.	
Mechanical alloying	Avoids the high temperature melting and solidification process of standard metallurgical methods, achieves alloying at room temperature and obtains uniform alloy with fine structures and high yields. Forms a uniform solid solution or compound.	[29,30,31,32,33,34,35]
Additive manufacturing	Produces arbitrary complex shape parts quickly and accurately. Figures out the forming of different intricate structure components. Curtails the processing process and shortens the processing cycle significantly.	[32,36,37,38,39]

**Table 2:** Properties of titanium and its alloy implants.

Properties	Remarks of Properties of Implants	References
Elastic modulus	Affects the stress transfer at the i junction surface between the implant and bone to the surrounding bone. Avoids the “stress shielding” effect, resulting in bone atrophy and implant displacement.	[43,44,45,46,47,48,49,50,51]
Fatigue strength	Affects the service life of the implant.	[52,53,54,55,56,57,58,59]
Degradation	Affects osseointegration	[60,61,62,63,64,65,66,67,68]
	Avoids peri-implantitis etc.	
Promoting bone growth	Enhances the stability and long-term survival rate of implants.	[69,70,71,72,73,74,75,76,77,78,79,80,81,82,83,84,85,86,87,88,89,90,91,92,93,94]
Inhibiting bacteria	Reduces inflammation. Improves implant stability and long-term survival rate.	[95,96,97,98,99,100,101,102,103,104,105,106,107,108,109,110,111,112,113,114,115,116]
Osseoperception	Improves patients’ perception and happiness index. Ensures the long-term use quality of implants.	[117,118,119,120,121,122]

## Data Availability

No new data were created or analysed in this study. Data sharing is not applicable to this article.
